# Chitosan/Gamma-Alumina/Fe_3_O_4_@5-FU Nanostructures as Promising Nanocarriers: Physiochemical Characterization and Toxicity Activity

**DOI:** 10.3390/molecules27175369

**Published:** 2022-08-23

**Authors:** Narges Ajalli, Mehrab Pourmadadi, Fatemeh Yazdian, Hamid Rashedi, Mona Navaei-Nigjeh, Ana M. Díez-Pascual

**Affiliations:** 1 Department of Chemical Engineering, Faculty of Engineering, University of Tehran, Tehran 1417935840, Iran; 2 Department of Life Science Engineering, Faculty of New Science and Technologies, University of Tehran, Tehran 1439956191, Iran; 3 Pharmaceutical Sciences Research Center, Institute of Pharmaceutical Sciences (TIPS), Tehran University of Medical Sciences, Tehran 1417613151, Iran; 4 Department of Pharmaceutical Biomaterials, Medical Biomaterials Research Center, Faculty of Pharmacy, Tehran University of Medical Sciences, Tehran 1417614411, Iran; 5 Universidad de Alcalá, Facultad de Ciencias, Departamento de Química Analítica, Química Física e Ingeniería Química, Ctra. Madrid-Barcelona, Km. 33.6, 28805 Alcalá de Henares, Madrid, Spain

**Keywords:** drug delivery, cancer, chitosan, gamma alumina, Fe_3_O_4_, fluorouracil

## Abstract

Today, cancer treatment is an important issue in the medical world due to the challenges and side effects of ongoing treatment procedures. Current methods can be replaced with targeted nano-drug delivery systems to overcome such side effects. In the present work, an intelligent nano-system consisting of Chitosan (Ch)/Gamma alumina (γAl)/Fe_3_O_4_ and 5-Fluorouracil (5-FU) was synthesized and designed for the first time in order to influence the Michigan Cancer Foundation-7 (MCF-7) cell line in the treatment of breast cancer. Physico-chemical characterization of the nanocarriers was carried out using X-ray diffraction (XRD), Fourier-transform infrared spectroscopy (FTIR), vibrating sample magnetometry (VSM), dynamic light scattering (DLS), and scanning electron microscopy (SEM). SEM analysis revealed smooth and homogeneous spherical nanoparticles. The high stability of the nanoparticles and their narrow size distribution was confirmed by DLS. The results of the loading study demonstrated that these nano-systems cause controlled, stable, and pH-sensitive release in cancerous environments with an inactive targeting mechanism. Finally, the results of MTT and flow cytometry tests indicated that this nano-system increased the rate of apoptosis induction on cancerous masses and could be an effective alternative to current treatments.

## 1. Introduction

Today, on hearing the name of cancer, a person becomes frightened and worried and loses hope in life. Generally, different kinds of cancer are caused by the abnormal proliferation of cells in the body. Millions of cells are grouped to make different tissues and organs of the human body. The genes of each cell give it the necessary instructions, some of which are vague, so the cell behaves abnormally, causing the circulation of a group of abnormal cells in the blood or immune system with the potential to turn into a malignant tumor or cancer [1,2,3]. The body’s cells die during a regulated process and are replaced by new cells. Sometimes this normal process is dysregulated and the worn cells do not disappear, forming a mass that can turn into a tumor and become malignant or cancerous [4,5,6]. There are several ways to treat this dangerous disease. Surgery, as one of the treatment methods, faces drawbacks such as slowed down digestion [7]. It can also affect the patient’s eating behaviors. Chemotherapy is one of the most common methods for cancer treatment. It is based on slowing down or stopping the development of fast-growing cancer cells. However, it also damages intact body cells, which in turn causes other side effects [8,9,10,11]. Radiotherapy is another cancer treatment method. However, since it damages cancer cells, it harms healthy cells as well. It may also cause nutritional problems [12,13,14,15]. Finding a treatment with minimal side effects, reduced cost and minimal impact is a challenging task in the medical field. Therefore, scientists recently developed new methods for cancer treatment using nanoscience. Nowadays, nanoscience is gaining considerable attention in various fields of medicine, including cancer treatment. Nanotechnology has been utilized for the diagnosis and treatment of diseases through the use of nanoparticles and the development of targeted drug delivery methods [16,17,18,19,20,21,22].

Nanoparticles can be used for therapeutic purposes due to their unique properties, including high surface-to-volume ratio and nanometer size, comparable to that of the wavelength of light. They can be combined with several support components as well as pharmaceutically active compounds due to their larger size compared to traditional chemotherapeutic agents or biological macromolecular drugs. These dissolution components facilitate protection against degradation, imaging, targeting, and stimulus-based activation. On the other hand, the processing of nanoparticles in the body is different from that of traditional drugs. Nanoparticles exhibit unique hydrodynamic properties and bio-distribution profiles. It is worth noting that interactions taking place at the nano–bio level can be used for improved drug delivery [16,22].

Polymer nanoparticles are made of biodegradable and biocompatible polymers as pharmaceutical carriers. In recent years, their biodegradable nature has received considerable attention as potentially suitable systems for drug delivery due to their ability to release drugs gently and to load large amounts of drugs and prevent their degradation. In these systems, the drug is loaded or linked by a covalent bond to the polymer matrix. Polymer nanoparticles are used to improve surface quality and increase drug adsorption efficiency. In the pharmacotherapeutical treatment of cancers, drugs can be selectively delivered to suitable sites through the development of controlled drug delivery techniques. The potential use of nanoparticles as drug carriers (so-called nano-carriers) was identified as an important challenge over the past few years. Nano-carriers increase drug absorption. Frequently, drug resistance leads to the failure of anti-cancer drugs during cancer treatment. Many experts recommend using several drugs simultaneously [23,24,25,26,27,28].

Chitosan (Ch) is one of the most common biopolymers, with high potential for chemical modification to attain new properties and to produce nanomedicines in the biomedical field. Ch has interesting properties such as mucosal adhesion, in situ gelling, a cationic nature, antimicrobial action, controlled drug release, and permeation ability due to its primary amine groups. Ch nanoparticles are common choices as drug carriers because of unique properties including biocompatibility, low toxicity, biodegradability, and the high loading capacity for proteins, oligosaccharides and so forth. Moreover, chemical modifications of this biopolymer have improved its solubility in aqueous media, which further increases its biological activity and applications [29,30,31,32,33,34,35]. For example, a study conducted by Zaki et al. [36] showed that due to its unique properties, Ch could be very effective in the field of cancer treatment. The study revealed that it could encapsulate chemotherapy drugs and increase their solubility, reducing side effects, and could yield better therapeutic efficacy by controlling the release rate.

Aluminum oxide is an inorganic compound with the chemical formula Al_2_O_3_; it is an important amphoteric oxide and is available under a variety of brands, such as alumina and corundum. At the nanoscale, alumina is quasi-spherical and has two phases, namely, alpha and gamma, the second (γAl) being more stable. With high surface area and thermal stability, γAl nanoparticles can be transformed into a micro-porous spherical structure or a honeycomb structure of catalysts. Their unique features and properties include amphoterism, high catalytic activity, good stability, high melting point and hardness. In addition, these nanoparticles have phase stability when dispersed in water, which makes them a good choice to be used in the field of drug delivery [37,38,39,40].

A recent study by Fasihi et al. [41] on Al_2_O_3_ nanoparticles containing 5-Fluorouracil (5-FU) showed that they reduced the side effects of 5-FU and also inhibited cell growth and migration, and hence have great potential to be used for the treatment of colorectal cancer. On the other hand, the literature review conducted by Denes et al. [42] corroborates the well-known biocompatibility of alumina. In this study, articles on the biocompatibility of this inorganic compound according to the ISO 10993-1 International Standard were examined. In vitro and in vivo tests in animals and humans did not show any abnormal response to each biological effect mentioned in the standard, such as cytotoxicity, sensitization, implantation and genotoxicity. In another study developed by Anaya et al. [43], γAl was modified with long-chain carboxylic acid surface nanocrystals, and biocompatible polysulfone/γAl nanocomposites with high performance were reported for the first time. The results showed that some fatty acids were chemically adsorbed on the γAl surface and formed nanosized self-assembled structures with thermal transitions at high temperatures, approximately 100 °C above the melting temperature of the pure acids, which were shifted by approximately 50 °C in the presence of polysulfone. The chemistry involved in the adsorption was mild and green, complying with strict biosanitary protocols for biocompatible devices. In addition, in a different work [44], biocompatible alumina ceramics were evaluated for total replacement of the hip joint, and were reported to be ideal materials for medical applications such as implants owing to their biocompatibility and outstanding wear resistance. A brief review of the types of alumina used in total hip replacement, the development of medical-grade alumina, and the methods for in vivo and in vitro evaluation of alumina prostheses was presented [44]. Furthermore, important results were obtained regarding the biocompatibility of alumina and calcium hex aluminate under different conditions based on studies developed by Arbex et al. [45]. To confirm the biocompatibility of these materials, their biological function, i.e., their interaction with the organism, was investigated. Preliminary biocompatibility tests were performed via cytotoxicity evaluation. The results of such study showed that both washed and unwashed alumina were non-cytotoxic and viable, and hex aluminate revealed slight cytotoxicity under different experimental conditions. In another study, to improve the mechanical and biological properties of alumina NPs, these were combined with titanium diboride (TiB_2_) micro powder. The properties of Al_2_O_3_–TiB_2_ nanocomposite coatings with different weight percentages of TiB_2_ (20, 30 and 40% *w*/*w*) were studied experimentally. Cytotoxicity was also investigated, in particular that of Al_2_O_3_-30 wt% TiB_2_ as the optimal coating, and the results confirmed its non-toxicity and biocompatibility [46]. It has also been demonstrated [47] that carbon-doped alumina is more biocompatible than alumina alone, and it also has improved wear resistance and a lower surface grain exit percentage. Confocal images and Alamar blue reduction assay showed good cell attachment and proliferation of human bone marrow-derived mesenchymal stromal cells (hBMSCs). Bone gene and protein expression indicated that Al_2_O_3_/C had higher osteogenic potential than Al_2_O_3_. Consequently, the new Al_2_O_3_/C nanocomposite had improved mechanical and biological properties. These findings demonstrated that the Al_2_O_3_/C nanocomposite had better performance than Al_2_O_3_ and therefore has more potential to be used in orthopedic applications [47].

Iron oxide magnetic nanoparticles (Fe_3_O_4_ MNPs) have attracted interest for different applications such as magnetic resonance imaging (MRI), targeted drug delivery, tissue repair, tumor targeting, cell isolation, and hyperthermia due to outstanding properties including nanoscale size, low toxicity, superparamagnetism, and catalytic activity [16,48,49]. At present, several methods including co-precipitation, hydrothermal, and sol–gel are used to synthesize these MNPs. Co-precipitation is a suitable approach to produce highly stable MNPs with an ultrafine size distribution, which results in improved magnetic properties. It is believed that toxicity is reduced by coating the surface of the MNPs with a polymer layer or by producing two hydroxides. Moreover, biocompatibility may be increased and aggregation avoided due to the adsorption of coating agents on the MNP surface. On the other hand, biocompatible compounds such as dextran, polyethylene glycol, polyvinyl alcohol, and polyvinylpyrrolidone have been used for surface modification or coating of MNPs to regulate their properties. The polymer layer applied to modify the MNP surface should be protein-resistant and non-antigenic to increase blood circulation time [50,51,52,53,54].

Noticeable efforts have focused on studying the effects of coating materials on the behavior of MNPs. In a study conducted by Nafchi et al. [55], Fe_3_O_4_ nanoparticles were functionalized by glutaric acid and then combined with carbon quantum dots. Subsequently, they were loaded with doxorubicin to treat breast cancer. The physico-chemical properties and morphology of the nanoparticles were examined, and the results showed that the synthesized nanocomposites could be used in MRI, targeted drug delivery, and cell labeling [55]. Chen et al. [56] showed that Fe_3_O_4_ nanoparticles are biocompatible and have therapeutic effects in combination with magnetic fluid hyperthermia on MCF-7 cancer cells. In that study, Fe_3_O_4_ MNPs were prepared using the simultaneous deposition method. Blood toxicity, laboratory toxicity and genotoxicity were investigated, and therapeutic effects were assessed via MTT test and flow cytometry assay. The results revealed that the toxicity of the MNPs in mouse fibroblast cell lines (L-929) was between grade 0 and grade 1 and that they had no hemolytic activity. Acute toxicity (LD50) was 8.39 g/kg. The micronucleus test showed no genotoxic effect. Path morphology and blood biochemistry testing showed that Fe_3_O_4_ nanoparticles had no effect on the main organs and blood biochemistry in the rabbit model. MTT assay and flow cytometry disclosed that Fe_3_O_4_ nano-magnetofluid heat therapy inhibited MCF-7 cell proliferation and its inhibitory effect was dose-dependent according to Fe_3_O_4_ nano-magnetofluid concentration [56]. In addition, in a study developed by Ankamwar et al. [57], the biocompatibility of Fe_3_O_4_ nanoparticles was evaluated by in vitro cytotoxicity assay using normal, glia, and breast cancer cells. The results of such research revealed that Fe_3_O_4_ nanoparticles coated with dipolar surfactant tetramethylammonium 11-aminoundecanoate were biocompatible and promising for biological applications such as drug delivery, magnetic resonance imaging and magnetic hyperthermia [57]. A study was performed by Li et al. [58] to investigate the biocompatibility of Fe_3_O_4_@Au composite magnetic nanoparticles in laboratory conditions. The results demonstrated that these modified MNPs were highly biocompatible and safe, and hence are suitable for further application in tumor hyperthermia. In addition, the biocompatibility of empty Fe_3_O_4_ ferromagnetic nanoparticles (FNs) with human blood cells, red blood cells, white blood cells and platelets was investigated by Stamopoulos et al. [59]. For this purpose, FNs at a concentration of 19 mg/mL were dispersed in human blood samples subjected to a specific maturation process of mild incubation for 120 min at 20 °C. The morphology and specific surface characteristics of the blood cells were analyzed using optical microscopy (OM) and atomic force microscopy (AFM), in order to obtain information from the micrometer to the nanometer level. The results obtained from both techniques led to the same conclusion of high biocompatibility of the Fe_3_O_4_ FNs, even at the high concentrations investigated in this work. Thus, no blood cell destruction or significant side effects were observed due to the presence of Fe_3_O_4_ FNs [59].

**Scheme 1:**
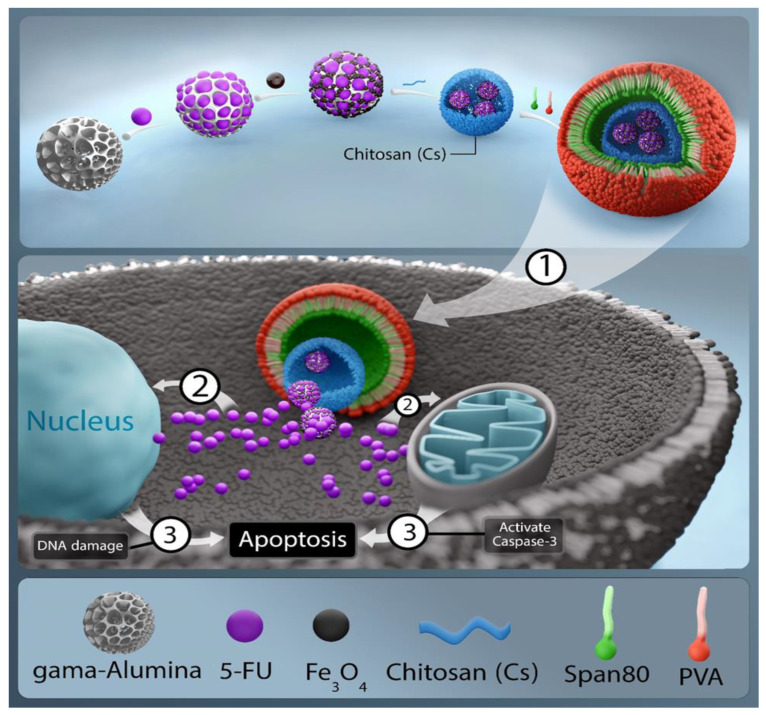
Formation of a 5-FU-loaded Chitosan/Gamma-Alumina/Fe_3_O_4_ hydrogel nanocomposite as nanocarrier with induction of apoptosis and DNA damage in the MCF-7 cell line.

Considering all the abovementioned studies, in the present work, (Ch)/γAl/Fe_3_O_4_ nanocomposites loaded with 5-FU were synthesized for the first time using specific methods at different temperatures. To the best of our knowledge, the mentioned nanoformulation led to the design of highly effective nanoplatforms for breast cancer treatment, as revealed by MTT assay and flow cytometry studies. These new nanocomposites increased the effectiveness of cancer treatment by reducing the toxicity and side effects of the pH-sensitive drug through its slow and controlled release. A general depiction of the experimental approach is shown in Scheme 1.

Span 80, a non-ionic surfactant with the chemical formula of C_64_H_124_O_26_, was used as an emulsifier. It is a transparent, odorless viscous liquid, an environmentally-friendly compound based on a natural fatty acid (oleic acid) and sorbitol sugar alcohol with unique hydrophilicity/hydrophobicity properties. The presence of sorbitan ester is very effective as a stabilizing agent in water-in-oil emulsion systems. This property is especially useful when used in combination with ethoxylated derivatives. Because of its biodegradable nature, its toxicity is very low. It is soluble in water, ethanol, methanol, isopropanol, toluene, ethyl acetate, and ether, while insoluble in acetone. Moreover, this material is stable in the presence of most chemical compounds and heat and oxidizes only in the presence of strong oxidants, and hence does not require special storage conditions. Such unique properties were the basic motivation for the use this material. In addition, polyvinyl alcohol (PVA), a synthetic polymer with low toxicity and high flexibility, was used in this study to prevent the adsorption or accumulation of Fe_3_O_4_ MNPs [60].

It is important to mention that Fluorouracil is one of the most important anti-cancer drugs, and it is increasingly used in chemotherapy for the treatment of various types of cancer due to its extensive anti-tumor activity as well as its ability to combine with other anti-cancer drugs. It has an anti-neoplastic effect and inhibits the synthesis of DNA and RNA. Its effectiveness is highly dependent on intracellular activation, and its applications are limited because high doses are required to attain good efficacy due to its nonspecific function and low plasma half-life, which leads to systemic toxicity in the body [61,62,63,64,65,66,67].

## 2. Materials and Methods

### 2.1. Materials

Urea, absolute ethanol, acetic acid (99.5%), and Span 80 were provided by Merck (Darmstadt, Germany). Sulfuric acid (98%), potassium permanganate, dialysis bag (12 kD cut off), hydrogen peroxide, annexins V- fluorescein isothiocyanate (FITC), Propidium Iodide (PI), 3-(4,5-dimethylthiazol-2-y1)-2,5-dipheny1 tetrazolium bromide (MTT) Powder, and 5-FU were obtained from Sigma Chemical Co. Olive oil was purchased from Niri Organic products (Tehran, Iran). A 1% penicillin/streptomycin solution, phosphate-buffered saline (PBS), RPMI 1640 and Dulbecco’s modified Eagle’s medium (DMEM) as culture media were supplied by INOCLON (G. Innovative Biotech Co, Tehran, Iran). Fetal bovine serum (FBS) was provided by Biochrome (Darmstadt, Germany). MCF-7 human breast cancer was obtained from the Research Institute of Biotechnology (Mashhad, Iran).

### 2.2. Synthesis of γAl

The sol–gel method was used to synthesize the γAl. Firstly, 3.9 H_2_O and 35 g of Al(NO_3_) deionized in 35 mL of distilled water were mixed at 22 °C using a magnetic stirrer. In a second step, 72 g of urea was added to the mentioned mixture and held for 1 h. In a third step, impurities were removed using a filter and the saturated aluminum urea solution was heated at 90 °C for approximately 12 h. Alumina sol was prepared by adjusting the pH of the solution to 2. Then, the solution was heated for 3 h to produce a gel. Finally, fresh alumina gel was heated in air at 280 °C for 1 h to remove impurities including urea and nitrate residues, resulting a porous gamma alumina nano-powder [68].

### 2.3. Synthesis of Ch/γAl Nano-Carriers

Firstly, 1 g of Ch was added to 50 mL of 2% acetic acid, and completely dissolved at room temperature, leading to a homogeneous gel-like solution of 2% Ch. Then, 50 mg of the γAl powder prepared in the previous step was added to the hydrogel until it was completely dissolved, and the solution was placed in an ultrasonic bath for 10 min [68,69].

### 2.4. Synthesis of Ch/γAl/Fe_3_O_4_ and Drug (5-FU) Loading Procedure

**Scheme 2:**
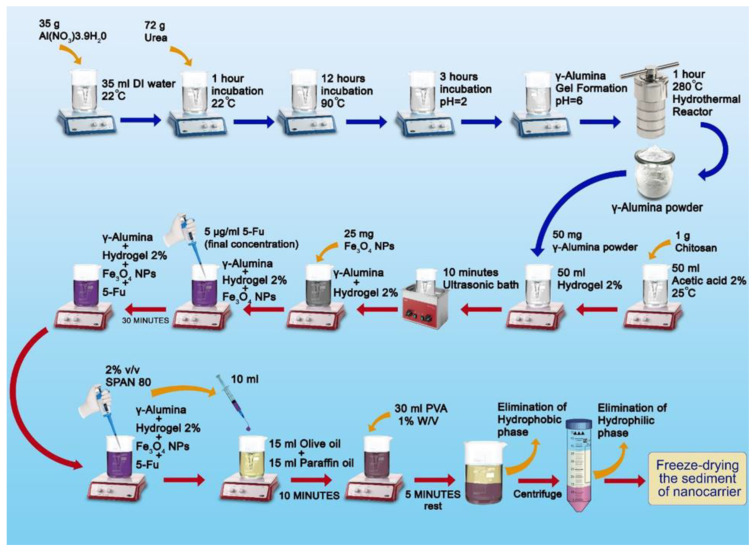
Synthesis method for drug-loaded Chitosan/Gamma-Alumina/Fe_3_O_4_ nanocomposites.

In this step, 25 mg of Fe_3_O_4_ nanoparticles was added to the solution and then homogenized in a heating stirrer. Subsequently, 5-FU drug was loaded, and the concentration of the drug in the final hydrogel was 5 μg/mL. The resulting mixture was placed on a heating stirrer for 30 min, and a uniform Ch/γAl/Fe_3_O_4_ hydrogel containing 5-FU was obtained. Span 80 surfactant (2% *v*/*v*) was smoothly added to the hydrogel under constant stirring. Next, 10 mL of Ch/γAl homogeneous hydrogel containing 5-FU coated with the surfactant was added dropwise to a hydrophobic phase consisting of 15 mL olive oil and 15 mL oily paraffin under stirring to form a spherical drug-containing nano-carrier in the hydrophobic phase. After 10 min, 30 mL of polyvinyl alcohol (PVA) (1% *w*/*v*) was added dropwise to the solution under stirring. The resulting suspension was removed from the stirrer and left for 5 min, so that the layers could be separated, and then the suspension was centrifuged at 600 rpm for 10 min. Finally, the nanoparticles were stored in a refrigerator at −20 °C, then transferred for freeze-drying [70,71,72,73,74,75,76]. The developed method for the synthesis of drug-loaded Chitosan/Gamma-Alumina/Fe_3_O_4_ nanocomposites is shown in Scheme 2.

### 2.5. Drug Loading and Encapsulation Efficiency

To calculate the content of 5-FU in the nano-carriers, 1 mg of the frozen nano-system dispersed in 1 mL of PBS solution (pH = 7.4) was added to the solution followed by 1 mL of ethyl acetate. Separately, the unloaded drug was dissolved in ethyl acetate as a free drug via stirring the solution. Then, the ethyl acetate phases were isolated and the absorbance of the dissolved drug was measured using a spectrophotometer at 266 nm. Equations (1) and (2) were used to estimate the amount of drug loaded and trapped for both nano-carriers, respectively, with regard to the initial and free amounts of the drug [77,78,79].

(1) $$\begin{array}{c} loading\;Efficiency\left(\%\right)\\ \;\;\;\;\;\;=\frac{\left(Total\;amount\;of\;5-FU\;\right)-\left(Free\;amount\;of\;5-FU\;\right)\;}{Total\;amount\;of\;nanocomposite}\times 100 \end{array}$$

(2) $$\begin{array}{c} Entrapment\;Efficiency\;\left(\%\right)\\ \;\;\;\;\;\;=\frac{\left(Total\;amount\;of\;5-FU\right)-\left(Free\;amount\;of\;5-FU\right)}{Total\;amount\;of\;5-FU}\times 100 \end{array}$$

### 2.6. Characteristic Assessment of Nano-Systems

The presence of functional groups in the different nanocomposite components as well as the interactions between the polymers, Ch/γAl/Fe_3_O_4_, and the drug in the nanocomposites was investigated using Fourier-transform infrared spectroscopy (FTIR) analysis (Thermo, AVATAR, America). KBr pellets of the samples, including Ch, γAl, Ch/γAl, Ch/γAl/Fe_3_O_4_, and Ch/γAl/Fe_3_O_4_@5-FU, were prepared, and their spectra were recorded in the range of 400 to 4000 cm^−1^. The amorphous and crystalline nature of samples in powder form was studied via X-ray diffraction (XRD) analysis, which also provided information on the crystalline phases of the materials and their unit cell dimensions. Diffractograms were recorded at 25 °C using a diffractometer (PHILIPS, PW1730, Amsterdam, The Netherlands). The surface morphology of the Ch/γAl/Fe_3_O_4_@5-FU nanocomposites was investigated using Scanning Electron Microscopy (SEM) analysis with a SEM microscope (TESCAN, MIRA III model, Czech Republic) after coating the samples with a gold overlayer to avoid charging during electron irradiation. Dynamic light scattering (DLS) analysis and zeta potential measurements at 25 °C were performed with a particle size analyzer (Horiba, SZ-100, Japan), which provided information regarding the size distribution of the drug-containing nanocarrier and its surface charge, respectively. A vibrating sample magnetometer (VSM) tool was used to characterize the magnetic properties of the nanoparticles. Cytotoxicity and cell apoptosis characterization of the nanocomposites was performed via MTT and flow cytometry assay, respectively.

### 2.7. In Vitro Study of Drug Release

The membrane diffusion technique was used to evaluate drug release from the nano-carriers. For this purpose, 1 mL of the nano-carrier solution containing the drug was poured into a dialysis bag with both ends tightly closed with a thread. Four dialysis bags were prepared, and each of them was immersed in a 15 mL PBS solution containing ethanol (20% *v*/*v*) at two different pHs (7.4 and 5.4); then, they were placed on a heating stirrer to keep the temperature at 37 °C. After 0, 12, 24, 48, 72, and 96 h, 300 µL of the solution was sampled and replaced by the same volume of fresh buffer. The light absorption of the drug in the samples was measured separately at a wavelength of 266 nm using a UV–Vis spectrophotometer, and then the concentration of drug released at each time was calculated using a standard curve, the so-called release diagram. The rate of drug release from the nano-carriers was calculated using Equation (3) [77,80].

(3) $$\mathrm{Drug}\;\mathrm{Released}\;\left(\%\right)=\frac{\left[drug\;\right]\;rel}{\left[drug\right]\;load}\times 100$$

### 2.8. Kinetic Modeling of Drug Release

- Zero-order model

In this kinetic model, the substance release takes place as a function of time. The process is performed at a constant speed and is independent of the substance concentration. The model can be used for a variety of drug delivery systems, including skin systems, slow-release matrices, osmotic systems, and coated systems. Equation (6) shows the relationship between time and the amount of drug released in this model [81]:

(4) $$W_{0}-W_{i}=k\times t$$

(5) $$1-\frac{W_{i}}{W_{0}}=k\times t\;\;\;\;\;$$

(6) $$\frac{W}{W_{0}}=K\times t\;\to f_{t}=K_{0}\times t$$

where *K*_0_, $f_{t}$, *W*, *W*_i_, *W*_0_, and *t* are the initial amount of drug in the carrier, the remaining amount of drug in the carrier at a given time, the cumulative amount of drug released at a given time, the deduction in the cumulative amount of drug released at a given time, fixed apparent dissolution rate, and time, respectively.

- First-order model

This semi-experimental kinetic model relates concentration changes over time to concentration; this means that the rate of concentration change in the carrier, and consequently in the environment, is a function of the amount of drug remaining in the carrier and, therefore, decreases over time. The first-order model is suitable for describing the release of soluble active substances from porous matrices [82].

(7) $$1-\frac{f_{t}}{f_{t,max}}=e^{-k_{1}t}$$

(8) $$\ln\left(1-\frac{f_{t}}{f_{t,\;max}}\right)=-k_{1}\;t\;$$

In Equation (8)$,f_{t}$, $f_{t,\;max}$, *K*_1_, and *t*, are the fraction of drug released at a given time, the maximum fraction of drug released during the process, the first-order rate constant, and time, respectively [83].

- Higuchi model

The Higuchi model is the most famous and widely used mathematical model to describe the release of drugs from matrix systems. In the case of homogeneous matrices, the drug release is controlled and limited by the penetration of solutes into the matrix, and the release mechanism follows the penetration. The amount released can be calculated according to the following equation [84]:

(9) $$\frac{M_{t}}{M_{\infty }}=k\times \sqrt{t}$$

where $M_{\infty }$ is the cumulative amount of drug released at an infinite time, $M_{t}$ is the cumulative amount of drug released at a given time (*t*), and *K* is a constant that includes different variables of the system design.

- Baker model

This model describes the behavior of controlled drug release from spherical matrices. The parameters of this equation are similar to those of the Higuchi equation [85].

(10) $$\frac{2}{3}\left(1-{\left(1-\frac{M_{t}}{M_{\infty }}\right)}^{\frac{2}{3}}\right)-\frac{M_{t}}{M_{\infty }}=k\times t$$

- Weibull model

The model was first proposed by Weibull and then used by Langenbushar to describe release curves. It is useful to compare the release profiles in matrix systems.

(11) $$m=1-e^{\left(\frac{-{\left(t-T_{i}\right)}^{b}}{a}\right)}$$

In this model, *a* is a parameter that indicates the time scale of the process, $T_{i}$ is a localization parameter that determines the latency of the propagation process, b is a shape parameter that specifies the type of rectifier for exponential, sigmoid and parabolic curves (considered equal to one, more than one, and less than one, respectively), and m is the cumulative fraction of the released drug.

Since the cumulative amount of drug released over time was exponential for the Ch/γAl/Fe_3_O_4_@5f-FU nano-system, *b* was considered equal to one. Thus, Equation (11) becomes [85]:

(12) $$m=1-e^{\left(\frac{-\left(t-T_{i}\right)}{a}\right)}$$

(13) $$\ln\left(1-m\right)=\frac{T_{i}}{a}-\frac{t}{a}$$

- Korsmeyer–Peppas model

This comprehensive and simple artificial model is used to express release behavior in polymeric systems:

(14) $$f_{1}=\frac{M_{i}}{M_{\infty }}=k\times t^{n}$$

(15) $$\ln\left(f_{1}\right)=\ln\left(K\right)+n\times \ln\left(t\right)$$

where *f*_1_, *M_i_*, $M_{\infty }$, *K*, and *n* are the fraction of released drug, the amount of drug released at time *t*, the amount of drug released under an equilibrium state that is sometimes very close to the initial amount of drug in the carrier, the release rate constant, and the release exponent, respectively. Drug delivery systems can be categorized based on the *n* parameter according to Table 1 [85].

- Hixson–Crowell model

This model describes a system in which changes in the surface and diameter of the drug carrier occur, and is expressed by Equation (16):

(16) $$\sqrt[{3}]{1-\frac{M_{t}}{M_{0}}=}\;1-k_{\beta }\times t\;$$

where *M_t_* is the amount of drug released at time *t*, *M*_0_ is the initial amount of drug in the carrier and *k_β_* is the rate release constant of the model [85].

### 2.9. MTT Test

The MTT test was used to measure cellular metabolic activity as an indicator of the degree of cytotoxicity. This colorimetric assay is based on the reduction of a yellow tetrazolium salt (3-(4,5-dimethylthiazol-2-yl)-2,5-diphenyltetrazolium bromide or MTT), with formula C_18_H_16_BrN_5_S, forming purple formazan crystals. The viable cells contain NAD(P)H-dependent oxidoreductase enzymes, which reduce the MTT to formazan. This test can detect and estimate cell death or apoptosis when cellular metabolism declines [86,87].

### 2.10. Flow Cytometry Test to Measure Apoptosis and Necrosis

Flow cytometry was applied to evaluate the extent of apoptosis and necrosis in the treated and untreated cells (control). Propidium iodide (PI) and Annexin staining kits were used for this purpose. The cells were first treated with a concentration of 2.8 g/mL 5-FU and incubated for 48 h. Afterwards, the two groups of cells were transferred to separate falcons after trypsinization and the cells were washed with PBS solution after centrifugation. The cells were mixed in 100 µL of Annexin/PI buffer for 15 min at 25 °C. Cellular apoptosis and propidium iodide cell necrosis were measured via Annexin. The fluorescence of the cell suspension, after being diluted in a 500 μL incubation buffer, was read using Becton Dickenson (BD) at an excitation wavelength of 488 nm [88,89].

## 3. Results and Discussion

### 3.1. Characteristic Analysis

**Figure 1:**
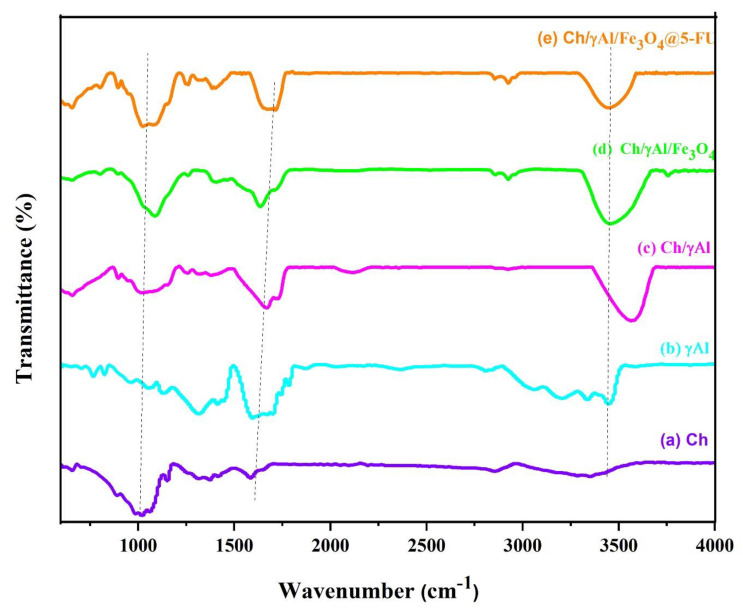
FTIR spectra of (**a**) Chitosan (Ch), (**b**) Gamma-Alumina (γAl,) (**c**) Chitosan/Gamma-Alumina (Ch/γAl), (**d**) Chitosan/Gamma-Alumina/Fe_3_O_4_ (Ch/γAl/Fe_3_O_4_), and (**e**) Chitosan/Gamma-Alumina/Fe_3_O_4_ @5-FU (Ch/γAl/Fe_3_O_4_@5-FU).

FTIR was used to identify Ch, γAl, Ch/γAl, Ch/γAl/Fe_3_O_4_, and Ch/γAl/Fe_3_O_4_@5-FU functional groups. The spectrum of neat Ch is shown in Figure 1a. The peaks at approximately 650 cm^−1^ are related to C–H bending vibrations. The peak at 887 cm^−1^ is assigned to out-of-plane bending of the C–H bond of the monosaccharide ring. The peaks appearing at 1020 cm^−1^ and 1060 cm^−1^ can be assigned to tensile vibration of the C–O bond, in agreement with the spectra recorded in previous articles [90,91,92,93,94]. The peak near 1320 cm^−1^ is attributed to the C–N tensile vibration of the ternary amide bond, which is consistent with the results from other articles [95]. The absorption peaks observed close to 1590 cm^−1^ are assigned to N–H bending of the primary amine groups [89], and those at 2510 cm^−1^ and 2856 cm^−1^ can be attributed to the symmetric and asymmetric stretching of the C–H bond, respectively. These are characteristic peaks of polysaccharides and have been identified in the spectra of xylene and glycan polysaccharides. The peak found at 3355 cm^−1^ is related to the tensile vibration of the O–H bond [96,97].

The spectrum of γ-Al is shown in Figure 1b. The peak at 603 cm^−1^ is attributed to the Al–O bending vibrations [98]. In amorphous samples, the tensile vibrations of Al–O(AlO_4_) cause a shift of these absorption peaks to 766 cm^−1^ and 824 cm^−1^ [99,100]. The peak at 964 cm^−1^ is related to the location of quadrilateral sites by Al^3+^ in the dense cubic structure of oxide ions in gamma phases [101]. The broad bands at approximately 3450 cm^−1^ belong to the tensile vibration of the O–H group attached to the Al^3+^ ions [58,79]. The peak appearing at 2800 cm^−1^ may be attributed to the C–H tensile vibration, indicating the presence of a small amount of urea, which is consistent with the observations reported by Elahi et al. [68]. The peaks in the range of 1140–2350 cm^−1^ are related to O–H bending vibrations, indicating the presence of hydration water [102,103,104,105].

The spectrum of Ch/γAl is shown in Figure 1c. The peak related to the O–H stretching appears at approximately 3566 cm^−1^, and is shifted towards higher wavenumbers compared to those of neat Ch and γAl. Similarly, the peaks related to the C–H stretching vibration of aliphatic bonds are shifted from 2856 cm^−1^ to 2931cm^−1^. The peak attributed to C–O stretching of the primary amide is also slightly shifted from 1650 cm^−1^ to 1658 cm^−1^. Likewise, the tensile vibration of the C–O bond of chitosan is shifted from 1020 cm^−1^ to 1018 cm^−1^ due to changes in the molecular interactions after the addition of γAl. These results are consistent with the studies by Zavareh et al. [106]. The peak at approximately 1319 cm^−1^ is assigned to C–N stretching vibrations of the ternary amide bonds. The presence of the characteristic peaks of chitosan in the Ch/γAl spectrum without significant changes in their wavenumber indicates that the incorporation of γ-Al into this biopolymer had hardly any effect on its structure. The peaks at 659 cm^−1^ and 550 cm^−1^ are attributed to Al–O bending vibrations. The most intense peaks of Al_2_O_3_ appear at 1658 cm^−1^ and 1384 cm^−1^. The bands in the range of 850–1018 cm^−1^ are ascribed to surface vibrations of alumina nanoparticles, caused by the loss of free alumina hydroxyl groups. These observations are consistent with the studies by Prakash et al. [107]. The aforementioned results corroborate the presence of Al_2_O_3_ nanoparticles in the nano-system membranes.

The spectrum of the Ch/γAl/Fe_3_O_4_ nanocomposite in Figure 1d shows two new peaks at 647 cm^−1^ and 590 cm^−1^ ascribed to the magnetic nanoparticles. In addition, a peak is found at 590 cm^−1^ related to the Fe–O tensile vibration. The absorption peak at 580 cm^−1^ is attributed to the Al–O vibration in the Ch/γAl/Fe_3_O_4_ composite. These results are consistent with the studies by Bozorgpour et al. [108]. The absorption peak at 470 cm^−1^ is attributed to quadrilateral and octahedral locations [109,110], and that appearing at 3356 cm^−1^ is due to the stretching vibration of O–H absorbed on the surface of the Fe_3_O_4_ nanoparticles. Chitosan coverage is confirmed by the peak appearing at 2923 cm^−1^, which corresponds to the –CH– starching vibration [111,112].

In the spectrum of Ch/γAl/Fe_3_O_4_ with 5-FU shown in Figure 1e, a new absorption peak can be found at 1698 cm^−1^, which corresponds to the vibrations of C=H bonds in CF=CH [90]. In addition, the absorption peaks at 1658 cm^−1^ and 1077 cm^−1^ are shifted to 1638 cm^−1^ and 1089 cm^−1^ [61,112,113].

Overall, the comparison of the FTIR spectra demonstrates that all nanocomposite components were present in the developed nano-carrier system. In addition, their interactions, and the interaction of the drug with the other components, were confirmed.

**Figure 2:**
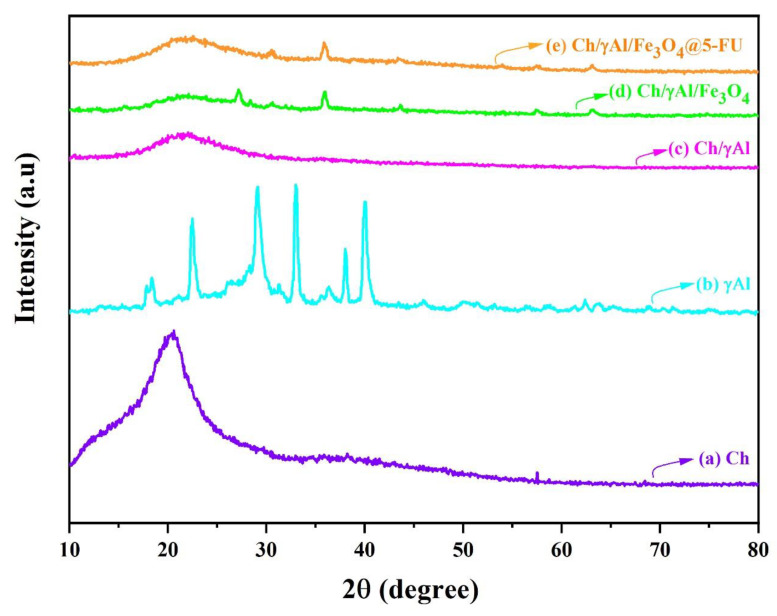
X-ray diffraction patterns of (**a**) Chitosan (Ch), (**b**) Gamma-Alumina (γAl,) (**c**) Chitosan/Gamma-Alumina (Ch/γAl), (**d**) Chitosan/Gamma-Alumina/Fe_3_O_4_ (Ch/γAl/Fe_3_O_4_), and (**e**) Chitosan/Gamma-Alumina/Fe_3_O_4_ @5-FU (Ch/γAl/Fe_3_O_4_@5-FU).

XRD analysis was performed for Ch, γAl, Ch/γAl, Ch/γAl/Fe_3_O_4_ and Ch/γAl/Fe_3_O_4_ containing 5-FU, and the diffractograms are displayed in Figure 2. The XRD pattern of Ch is shown in Figure 2a. A broad peak is found in the range of 2θ=20°−21°, indicating the hydrated crystalline structure of Ch. These results are consistent with those reported previously [94,114,115].

It is clear from Figure 2b that γ-Al has a crystalline structure. Intense peaks are observed at 2θ values of 33°, 40°, and 62°, which is consistent with the results reported by Nematollahi et al. [68]. The diffractogram of Ch/γAl in Figure 2c shows a broad peak centered at approximately 21°, significantly wider than that of neat chitosan. This indicates a reduction in the degree of crystallinity of Ch in the composite compared to that of the neat polymer, due to the addition of the γAl.

The effect of the addition of Fe_3_O_4_ MNPs to the nano-system is shown in Figure 2d. New peaks were observed at 2θ values of 26.9°, 30.35°, 35.65°, 43.3°, 53°, 57.5°, and 62.7°. Fe_3_O_4_ peaks were identified based on information in the HighScore Plus software database and the standard range. These peaks confirm the presence of Fe_3_O_4_ in the Ch/γAl/Fe_3_O_4_ nano-system. The changes in the peak intensity indicate interaction and entanglement with other components. The peak intensity in the nanocomposite is reduced, indicating the formation of a nano-system with an amorphous structure [116,117,118]. The result of placing 5-FU in the nano-system is shown in Figure 2e. Pure fluorouracil has strong peaks at 2θ of 22°, 19°, and 16°, which indicates the crystalline nature of this drug. The sharpening of the wide peak in the diffraction pattern of Ch/γAl/Fe_3_O_4_ with 5-FU confirms the presence of the drug in the nano-system [119,120,121].

In general, the peaks in the spectrum of the nano-system are broadened, which indicates a decrease in the degree of crystallinity and its approximation to an amorphous state. Despite comprising crystalline compounds, the final nano-system can be regarded as an amorphous complex with suitable solubility.

**Figure 3:**
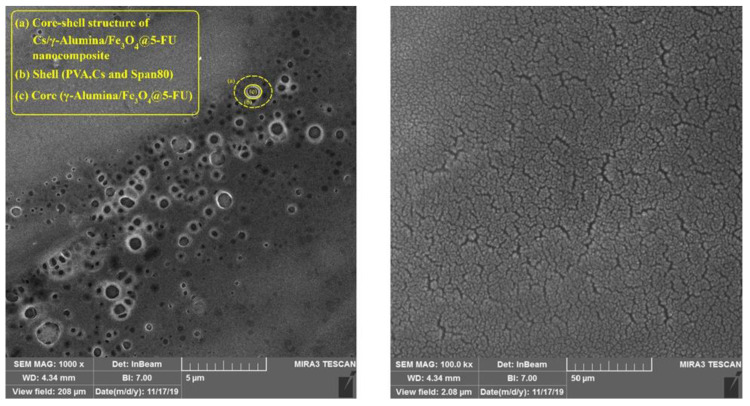
SEM images of Chitosan/Gamma-Alumina/Fe_3_O_4_@5-FU system. Scale bars are 5 μm (**left**) and 50 μm (**right**).

The morphology and size of the Ch/γAl/Fe_3_O_4_ nano-system along with 5-FU were assessed using SEM, and representative micrographs of the nano-system containing the drug are shown in Figure 3. The nano-systems were found to be solid and dense spherical structures, with an average diameter on the order of 300 nm. This spherical structure prevents the uncontrolled release of the drug and has high efficiency among drug delivery systems.

**Figure 4:**
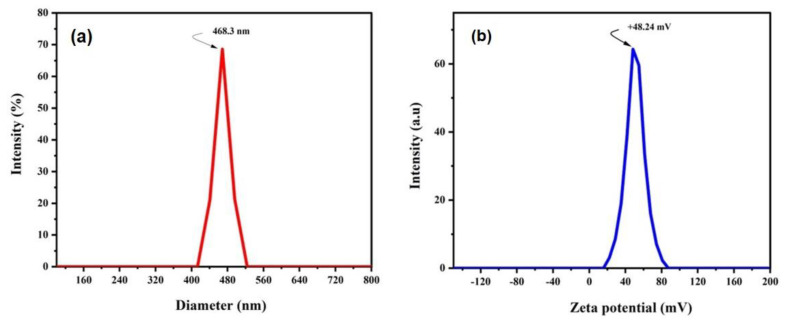
DLS and Zeta potential of Chitosan/Gamma-Alumina/Fe_3_O_4_@5-FU (Ch/γAl/Fe_3_O_4_@5-FU) nanocomposites.

DLS analysis was performed to characterize the size distribution of the Ch/γAl/Fe_3_O_4_ nano-carrier containing the drug. As shown in Figure 4a, the nanostructures were monodisperse with a mean hydrodynamic diameter of 468.3 nm, which is a good value for pharmaceutical applications [122,123,124]. As expected, this value was larger than that obtained from SEM analysis (Figure 3), since SEM measurements are carried out in the solid state while the hydrodynamic diameter is obtained from measurements of the diffusion coefficient of the nanoparticles in suspension. The hydrodynamic diameter gives information concerning the core particle, together with any coated material on the particle and the solvent layer, as it moves under the influence of Brownian motion. On the other hand, the PDI obtained from DLS analysis had a value of 0.5, indicative of a uniform particle size distribution. This parameter directly reflects the size homogeneity of the nanoparticles according to the abovementioned literature review.

The surface charge of the Ch/γAl/Fe_3_O_4_ nano-system containing the drug was determined via surface zeta potential measurements, which provide important information regarding the physical stability of nano-system and their interaction with cells and biomolecules [125]. If the suspended particles have a high positive or negative charge, they will repel each other and will not tend to accumulate. Figure 4b shows the zeta potential plot of the Ch/γAl/Fe_3_O_4_ nano-carrier containing the drug. The potential of the output device was + 48.24 mV, which confirms the good stability of the developed nano-system. The high zeta potential value of the nanoparticles is the result of the amine groups, which show proper chitosan coverage.

**Figure 5:**
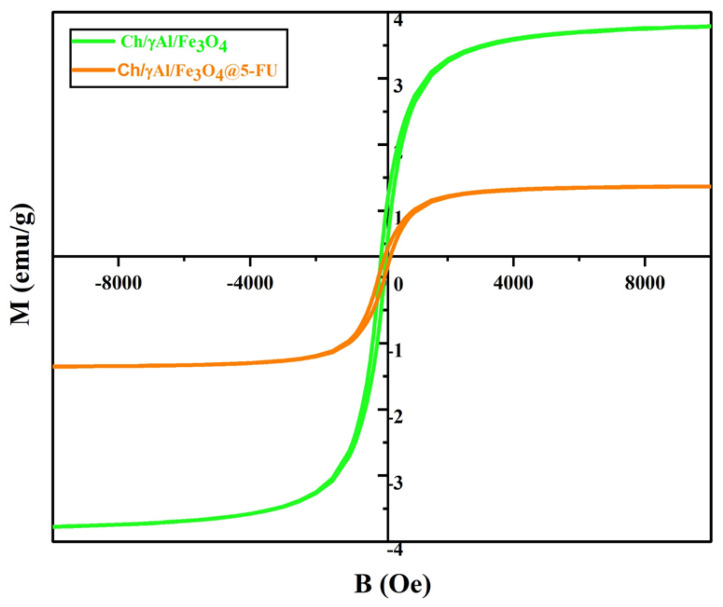
VSM result for Chitosan/Gamma-Alumina/Fe_3_O_4_ (Ch/γAl/Fe_3_O_4_) and Chitosan/Gamma-Alumina/Fe_3_O_4_@5-FU (Ch/γAl/Fe_3_O_4_@5-FU) nanoparticles.

VSM analysis was performed to measure the magnetic properties of the synthesized Ch/γAl/Fe_3_O_4_ nano-carrier as well as the Ch/γAl/Fe_3_O_4_ loaded with 5-FU. According to the results (Figure 5), no magnetic reversal cycle was observed. The magnetic properties of the nanoparticles decreased after coating with the chitosan shell due to the formation of a core skin structure with the non-magnetic polymeric chains on the surface. However, both Ch/γAl/Fe_3_O_4_ and Ch/γAl/Fe_3_O_4_@5-FU nanocomposites retained magnetic behavior. At the maximum applied field, their magnetic values were 3.8 emu/g and 1.4 emu/g, respectively. These results indicate that the proposed nanostructure could also act as a targeting system for anti-cancer drugs in the presence of an external magnetic field [76].

### 3.2. Percentage of Drug Loading and Encapsulation Efficiency Results

According to previous studies [76], a high load percentage of the drug can become attached to the porous structure of the nanoparticles, which can trap more molecules inside [76]. On the other hand, the addition of γAl to the Ch/Fe_3_O_4_ nano-system provides more and stronger surface interactions between the polymers, γAl and the drug, due to the high γAl content. In the present work, the loading and trapping efficiency of the anti-cancer drug were estimated to be 43% and 88.5%, respectively, and these values are acceptable for drug delivery applications.

### 3.3. In Vitro Evaluation of Drug Delivery

**Figure 6:**
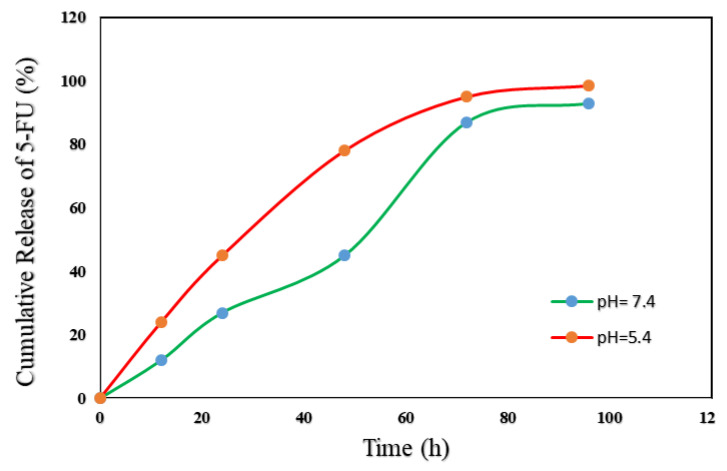
Cumulative 5-FU release from the developed nanocomposite versus time at pH 5.4 and 7.4.

The in vitro release of 5-FU from the developed nano-system was investigated to confirm the successful design of a pH-sensitive and stable nanocarrier for drug release. The release profile of 5-FU from the nano-carriers was obtained via dialysis in two buffer media at different pHs at 37 °C for 96 h [126]. According to Figure 6, 12, 27, 45, 87 and 93% of 5-FU were released from the Ch/γAl/Fe_3_O_4_ nano-carrier in an acidic environment (pH = 5.4) after 12 h, 24 h, 48 h, 72 h and 96 h, respectively, while the release percentages in the biological medium (pH = 7.4) were 24, 45, 78, 95, and 98.5%, respectively. These results evidence long-term release via stable diffusion for 96 h. The comparison of the values obtained at the two pHs indicates a higher amount of drug released at acidic pH compared to that at pH 7.4, which confirms the pH-sensitivity of this drug delivery system. Cancer cells, due to over-proliferation and lack of sufficient oxygen, move into anaerobic pathways and produce lactic acid at a pH lower than normal tissues. As a result, this designed drug delivery system has a controlled and specific release capability. The drug is inactivated in the acidic environment of cancer cells by the targeting mechanism, thereby reducing the toxic effects of the drug on non-cancer cells.

### 3.4. Modeling the Kinetics and the Mechanism of Drug Release

**Figure 7:**
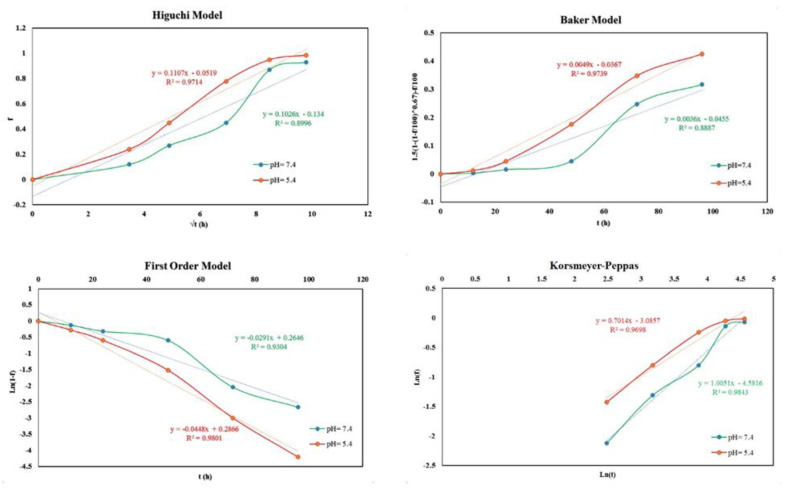

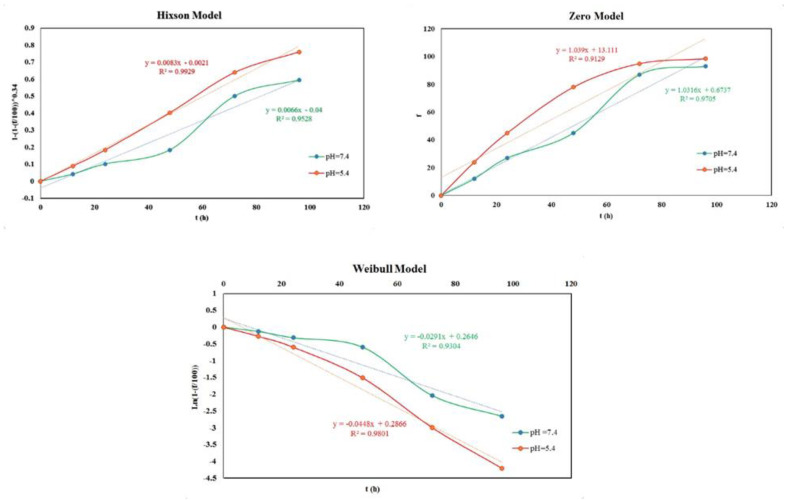
Drug release modeling.

Drug release data were analyzed with seven models including zero-order, first-order, Higuchi, Hixson–Crowell, Korsmeyer–Peppas, Baker, and Weibull to determine the drug release kinetics [94]. The fitting of the drug release data to the indicated models, their degree of correlation, and the values of n in the Korsmeyer–Peppas kinetic model that indicate the type of drug release mechanism are shown in Table 1 and Table 2. According to Figure 7 and Table 1, at a pH of 7.4, the Korsmeyer–Peppas model had the highest value of the R^2^ correlation coefficient. Therefore, it shows the best fit to the release profiles and indicates the release mechanism of the drug. Table 2 summarizes the values of n that can be obtained with this model and the corresponding release mechanisms. Given that the value of n obtained herein (0.43) was in the range 0.4 < *n* < 0.85, it can be concluded that the drug release from the prepared nano-carrier follows a polymer swelling mechanism and drug penetration and diffusion occur outside the structure of the nano-system. Under these conditions, the rearrangement of the polymer chains is slow and the diffusion process simultaneously causes unusual time-dependent effects.

On the other hand, according to Figure 7 and Table 1, the data obtained from the drug release profile at pH = 5.4 indicate that the Hixson–Crowell model, with a correlation coefficient of 0.9929, was the best to describe the release mechanism. Then, the first-order and zero-order kinetic models show the next highest R-squared values. In fact, according to Figure 7, at acidic pH values, the drug release from the prepared nano-carrier takes place under a controlled and continuous manner.

### 3.5. MTT Test

MTT testing was applied to examine the cytotoxicity of the samples. In this test, the toxicity of the free drug, Ch, Ch/γAl, blank Ch/γAl/Fe_3_O_4_ (without drug loading), and Ch/γAl/Fe_3_O_4_@5-FU incubated with MCF-7 cells after 72 h was investigated. An untreated control group was used to compare the cell survival of the abovementioned samples. An equivalent drug concentration of 5 μg/mL was used in all the drug-containing formulations.

**Figure 8:**
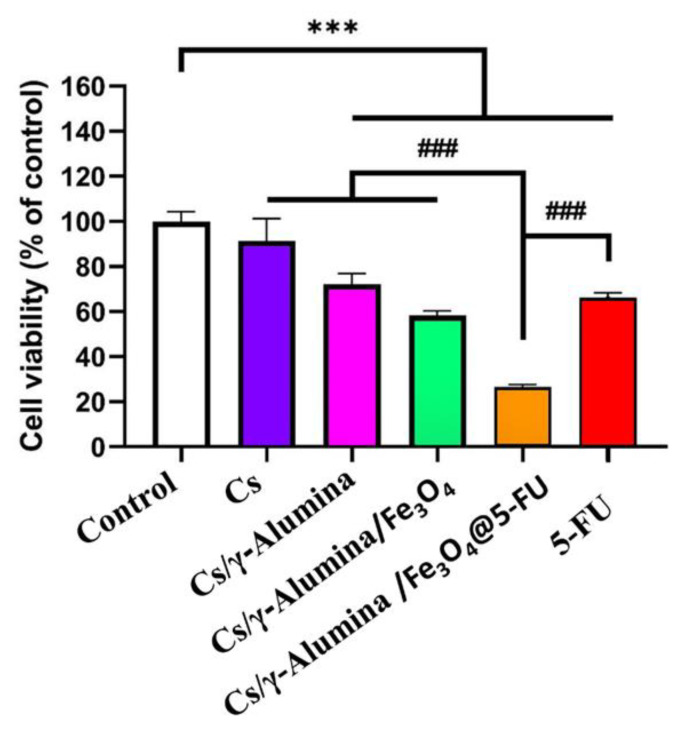
Comparison of in vitro cytotoxicity of Control, Chitosan, Chitosan/γ-Alumina, Chitosan/γ-Alumina/Fe_3_O_4_, Chitosan/γ-Alumina/Fe_3_O_4_@5-FU, and 5-FU (*** means *p*-value < 0.001 in comparison with Control, and ### means *p*-value < 0.001 in comparison to Chitosan, Chitosan/*γ*-Alumina/Fe_3_O_4_@5-FU with 5-FU).

The results showed that cell treatment with the free drug reduced cell survival compared to the control group. In addition, Ch/γAl/Fe_3_O_4_ decreased cell survival and had a statistically significant effect compared to the control group, indicating that these nanoparticles have anti-cancer properties. However, without 5-FU, it did not have a significant effect on cell death, which confirms the biocompatibility of these nanoparticles for drug delivery applications. Figure 8 shows the increasing effect of the anti-cancer drug 5-FU in the nanocomposite. The final drug-containing nanocomposite had the greatest effect in inhibiting the proliferation of cancer and inducting cancer cell death. From this result, combined with the targeted and controlled release of the drug, it can be concluded that the synthesized nanocomposite was able to increase the impact on cancerous masses along with reducing restrictions on the use of the free drug [126]. One of the possible mechanisms of the toxicity of the nanoparticles is the induction of reactive oxygen species and subsequent oxidative stress in tissues and cells. Therefore, testing the interaction of nanoparticles with proteins and different types of cells should be considered part of the toxicology assay. Except for aerosols delivered to the lungs, information on the biological fate of nanoparticles, including distribution, accumulation, metabolism, and specific organ toxicity, is still scarce [127]. γAl and Fe_3_O_4_, similarly to other nanoparticles, may induce toxicity, especially at high concentrations, and this should be considered in the toxicology assay. In order to decrease γAl toxicity, a biocompatible biopolymer (Ch) was used, which improved the cell viability and pH sensitivity of the developed nano-carriers via surface functionalization of the drug-loaded nanoparticles. In addition, double emulsion methods and span 80 as a surfactant were used to increase the stability and uniformity of the nanocarriers and to prevent the precipitation of the nanoparticles during delivery through the body [68,128]. In contrast, based on the MMT assay, 4.8 μg.ml^−1^ of the free drug led to IC50 on the MCF-7 cell line (more than 50% cell death) [126], which is consistent with our study.

### 3.6. Flow Cytometry Results

**Figure 9:**
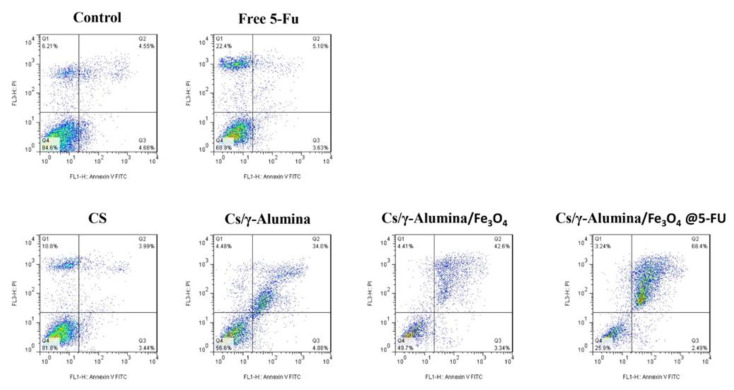
Cytotoxicity analysis of Control, Chitosan, Chitosan/γ-Alumina, Chitosan/γ-Alumin/Fe_3_O_4_, Chitosan/γ-Alumina/Fe_3_O_4_@5-FU, and free 5-FUtoward MCF-7 cells for 72 h.

Flow cytometry testing was used to evaluate the effect of the synthesized nanocomposite on the rate of apoptosis and cell necrosis. Figure 9 shows the outcome of the free drug, Ch, Ch/γAl, Ch/γAl/Fe_3_O_4_, Ch/γAl/Fe_3_O_4_ @5-FU and control groups on the apoptosis and necrosis of the MCF-7 cell line. The transfer of polar heads of serine phosphatide from the inner monolayer of the plasma membrane to its outer surface was considered an indicator of the early stages of apoptosis. Annexin V protein showed high binding affinity to phosphatidylserine, a phospholipid of the membrane. On the other hand, by increasing the permeability of the cell membranes in the delayed phases of apoptosis and necrosis, PI dye entered the cell.

Examination of the cultured control sample after the tests showed that 86.6% of the cells were in the living cell phase, 6.21% in the necrosis phase, 4.66% in the primary phase of apoptosis, and 4.55% in the secondary phase of apoptosis. The effect of the free drug on the cancer cells was investigated to compare with that of the synthesized nanocomposite. A substantial increase in the percentage of cells in the necrosis phase, which reached 22.4%, was found compared to the control group. Furthermore, a significant effect was evident on the apoptotic secondary phase cells with a 5.10% increase. Apoptotic cells in the early stages reached 3.63%. The developed nanocomposite increased the percentage of living cells to 25.9% and decreased that of necrotic cells to 3.24%. The effect was significant on the apoptosis-phase cells, and the primary and secondary apoptosis-phase cells reached 2.49% and 68.4%, respectively. These results demonstrate that this nanocomposite induced apoptosis at the highest rate. When the drug is released steadily over a long period and at an almost constant concentration, the cancer cell can enter the programmed death cycle; hence, the rate of apoptosis can be increased, since it is influenced by the drug release mechanism. The results demonstrate the high potential of the synthesized nanocomposite to increase apoptosis, or induce programmed death, in cancer cells and reduce malignancy, or sudden cell death, to prevent inflammation and reduce the destructive effects on non-cancerous cells, which are the ultimate goals of a drug delivery system [126].

## 4. Conclusions

Undoubtedly, cancer patients need new combination of therapies with better performance and minimal side effects. Selective and effective targeted drug delivery can direct chemotherapy agents to the target site, reducing systematic toxicity and improving the therapeutic effect. Therefore, in this study, a Ch/γAl/Fe_3_O_4_ nano-system was synthesized and characterized for the first time, and the anti-cancer drug 5-FU was loaded into the nanostructure. The physical, chemical, and magnetic properties of the nano-system were structurally and biologically analyzed through several tests. The morphology and size of the system containing 5-FU were studied using scanning electron microscopy images. The average diameter of the nano-carriers was on the order of 300 nm. The results from zeta potential analysis demonstrated the high stability of the developed nano-carriers. An increase in the percentage of drug loaded and trapped in the shell–core structure of the developed drug delivery systems was found when adding Fe_3_O_4_. The designed nano-systems also enabled controlled and pH-sensitive drug release in the environment, and did not produce any toxicity, which confirms the biocompatibility of the nanoparticles as drug carriers. Experimental results show that the developed nano-carrier could release 43% of the drug within the first 12 h. Therefore, with 65% cancer cell death, the controlled and targeted release provided by the developed nano-carrier reduced side effects and killed cancer cells selectively compared to non-targeted drugs. Overall, the nano-systems developed in this study could be used as efficient drug delivery systems for cancer treatment.

## Figures and Tables

**Table 1:** Release kinetics of 5-FU according to the different models.

Sample	pH	Zero-Order	First-Order	Higuchi	Korsmeyer	Hixson	Baker	Weibull
		R^2^	R^2^	R^2^	R^2^	R^2^	R^2^	R^2^
Ch/γAl/Fe_3_O_4_@5-FU	7.4	0.9705	0.9304	0.8996	0.9843	0.9528	0.8887	0.9304
	5.4	0.9129	0.9801	0.9714	0.9698	0.9929	0.9739	0.9801

**Table 2:** Classification of release mechanisms in the Korsmeyer–Peppas model based on the release exponent in different carrier geometries.

Release Mechanism Model	Geometry	Release Exponent (*n*)
Fickian diffusion	Planar	0.5
	Cylinders	0.45
	Spheres	0.43
Anomalous transport	Planar	0.5 < *n* < 1
	Cylinders	0.45 < *n* < 0.89
	Spheres	0.43 < *n* < 0.85
Case I transport	Planar	1
	Cylinders	0.89
	Spheres	0.85
Super Case II transport	Planar	*n* > 1
	Cylinders	*n* > 0.89
	Spheres	*n* > 0.85

## Data Availability

Data are included within this article.

## References

[1] Stone J.D., Harris D.T., Kranz D.M. (2015). TCR affinity for p/MHC formed by tumor antigens that are self-proteins: Impact on efficacy and toxicity. Curr. Opin. Immunol..

[2] Kopper L., Hajdú M. (2004). Tumor stem cells. Pathol. Oncol. Res..

[3] Intlekofer A.M., Finley L.W. (2019). Metabolic signatures of cancer cells and stem cells. Nat. Metab..

[4] Bergers G., Fendt S.-M. (2021). The metabolism of cancer cells during metastasis. Nat. Rev. Cancer.

[5] Icard P., Shulman S., Farhat D., Steyaert J.-M., Alifano M., Lincet H. (2018). How the Warburg effect supports aggressiveness and drug resistance of cancer cells?. Drug Resist. Updates.

[6] Waks A.G., Winer E.P. (2019). Breast cancer treatment: A review. JAMA.

[7] Pucci C., Martinelli C., Ciofani G. (2019). Innovative approaches for cancer treatment: Current perspectives and new challenges. Ecancermedicalscience.

[8] Loriot Y., Zoubeidi A., Gleave M.E. (2012). Targeted therapies in metastatic castration-resistant prostate cancer: Beyond the androgen receptor. Urol. Clin..

[9] Trudeau M., Charbonneau F., Gelmon K., Laing K., Latreille J., Mackey J., McLeod D., Pritchard K., Provencher L., Verma S. (2005). Selection of adjuvant chemotherapy for treatment of node-positive breast cancer. Lancet Oncol..

[10] Nygren P. (2001). What is cancer chemotherapy?. Acta Oncol..

[11] Ozols R. (2006). Challenges for chemotherapy in ovarian cancer. Ann. Oncol..

[12] Schaue D., McBride W.H. (2015). Opportunities and challenges of radiotherapy for treating cancer. Nat. Rev. Clin. Oncol..

[13] Chen H.H., Kuo M.T. (2017). Improving radiotherapy in cancer treatment: Promises and challenges. Oncotarget.

[14] Rosenblatt E., Zubizarreta E. (2017). Radiotherapy in Cancer Care: Facing the Global Challenge.

[15] Samiei M. (2013). Challenges of making radiotherapy accessible in developing countries. Cancer Control.

[16] Pourmadadi M., Ahmadi M.J., Dinani H.S., Ajalli N., Dorkoosh F. (2022). Theranostic applications of stimulusresponsive systems based on Fe_2_O_3_. Pharm. Nanotechnol..

[17] Ahmad I., Khan M.F.A., Rahdar A., Hussain S., Tareen F.K., Salim M.W., Ajalli N., Amirzada M.I., Khan A. (2022). Design and Evaluation of pH Sensitive PEG-Protamine Nanocomplex of Doxorubicin for Treatment of Breast Cancer. Polymers.

[18] Arshad R., Sargazi S., Fatima I., Mobashar A., Rahdar A., Ajalli N., Kyzas G.Z. (2022). Nanotechnology for Therapy of Zoonotic Diseases: A Comprehensive Overview. ChemistrySelect.

[19] Al-Joufi F.A., Setia A., Salem-Bekhit M.M., Sahu R.K., Alqahtani F.Y., Widyowati R., Aleanizy F.S. (2022). Molecular pathogenesis of colorectal cancer with an emphasis on recent advances in biomarkers, as well as nanotechnology-based diagnostic and therapeutic approaches. Nanomaterials.

[20] Alshareeda A.T., Khatijah M.N., Al-Sowayan B.S. (2022). Nanotechnology: A revolutionary approach to prevent breast cancer recurrence. Asian J. Surg..

[21] Wang T., Cao C., Gavahi M., Sadri M., Rong X. (2022). Using Nanotechnology for Diagnosis and Treatment of Breast Cancer: A Review. Indian J. Pharm. Sci..

[22] Yadav N., Dahiya T., Chhillar A.K., Rana J.S., Saini H.M. (2022). Nanotechnology in Cancer Diagnostics and Therapeutics: A Review. Curr. Pharm. Biotechnol..

[23] Jawahar N., Meyyanathan S. (2012). Polymeric nanoparticles for drug delivery and targeting: A comprehensive review. Int. J. Health Allied Sci..

[24] Raman S., Mahmood S., Hilles A.R., Javed M.N., Azmana M., Al-Japairai K.A. (2020). Polymeric nanoparticles for brain drug delivery—A review. Curr. Drug Metab..

[25] Leyva-Gómez G., Piñón-Segundo E., Mendoza-Muñoz N., Zambrano-Zaragoza M.L., Mendoza-Elvira S., Quintanar-Guerrero D. (2018). Approaches in polymeric nanoparticles for vaginal drug delivery: A review of the state of the art. Int. J. Mol. Sci..

[26] Kumari A., Yadav S.K., Yadav S.C. (2010). Biodegradable polymeric nanoparticles based drug delivery systems. Colloids Surf. B Biointerfaces.

[27] Nagavarma B., Yadav H.K., Ayaz A., Vasudha L., Shivakumar H. (2012). Different techniques for preparation of polymeric nanoparticles-a review. Asian J. Pharm. Clin. Res..

[28] Jahangiri A., Barghi L. (2018). Polymeric nanoparticles: Review of synthesis methods and applications in drug delivery. J. Adv. Chem. Pharm. Mater. JACPM.

[29] Shanmuganathan R., Edison T.N.J.I., LewisOscar F., Kumar P., Shanmugam S., Pugazhendhi A. (2019). Chitosan nanopolymers: An overview of drug delivery against cancer. Int. J. Biol. Macromol..

[30] Shafabakhsh R., Yousefi B., Asemi Z., Nikfar B., Mansournia M.A., Hallajzadeh J. (2020). Chitosan: A compound for drug delivery system in gastric cancer—A review. Carbohydr. Polym..

[31] Babu A., Ramesh R. (2017). Multifaceted applications of chitosan in cancer drug delivery and therapy. Mar. Drugs.

[32] Ghaz-Jahanian M.A., Abbaspour-Aghdam F., Anarjan N., Berenjian A., Jafarizadeh-Malmiri H. (2015). Application of chitosan-based nanocarriers in tumor-targeted drug delivery. Mol. Biotechnol..

[33] Prabaharan M. (2015). Chitosan-based nanoparticles for tumor-targeted drug delivery. Int. J. Biol. Macromol..

[34] Zhang K., Hocker J.D., Miller M., Hou X., Chiou J., Poirion O.B., Qiu Y., Li Y.E., Gaulton K.J., Wang A. (2021). A single-cell atlas of chromatin accessibility in the human genome. Cell.

[35] Narmani A., Jafari S.M. (2021). Chitosan-based nanodelivery systems for cancer therapy: Recent advances. Carbohydr. Polym..

[36] Ahmad M.Z., Rizwanullah M., Ahmad J., Alasmary M.Y., Akhter M.H., Abdel-Wahab B.A., Warsi M.H., Haque A. (2022). Progress in nanomedicine-based drug delivery in designing of chitosan nanoparticles for cancer therapy. Int. J. Polym. Mater. Polym. Biomater..

[37] Manzoor S., Ashraf M.W., Tayyaba S., Hossain M.K. (2021). Recent progress of fabrication, characterization, and applications of anodic aluminum oxide (AAO) membrane: A review. arXiv.

[38] Hassanpour P., Panahi Y., Ebrahimi-Kalan A., Akbarzadeh A., Davaran S., Nasibova A.N., Khalilov R., Kavetskyy T. (2018). Biomedical applications of aluminium oxide nanoparticles. Micro Nano Lett..

[39] Santos A., Kumeria T., Losic D. (2013). Nanoporous anodic aluminum oxide for chemical sensing and biosensors. TrAC Trends Anal. Chem..

[40] Ingham C.J., ter Maat J., de Vos W.M. (2012). Where bio meets nano: The many uses for nanoporous aluminum oxide in biotechnology. Biotechnol. Adv..

[41] Fasihi K., Amerizadeh F., Sabbaghzadeh R., Heydari M., Rahmani F., Mostafapour A., Khazaei M., Rasouli E., Hassanian S.M., Ferns G.A. (2022). The therapeutic potential of γ-Al_2_O_3_ nanoparticle containing 5-fluorouracil in the treatment of colorectal cancer. Tissue Cell.

[42] Denes E., Barrière G., Poli E., Lévêque G. (2018). Alumina biocompatibility. J. Long Term Eff. Med. Implant..

[43] Anaya S., Serrano B., Herrero B., Cervera A., Baselga J. (2014). γ-alumina modification with long chain carboxylic acid surface nanocrystals for biocompatible polysulfone nanocomposites. ACS Appl. Mater. Interfaces.

[44] Zeng P. (2008). Biocompatible alumina ceramic for total hip replacements. Mater. Sci. Technol..

[45] Arbex R., Rezende L., Ambrosio F., Costa L., Lombello C. Cytotoxicity of Alumina and Calcium Hexaluminate: Test Conditions. Proceedings of the XXVII Brazilian Congress on Biomedical Engineering, CBEB 2020.

[46] Alvar F.S., Heydari M., Kazemzadeh A., Vaezi M.R., Nikzad L. (2020). Synthesis and characterization of corrosion-resistant and biocompatible Al_2_O_3_–TiB2 nanocomposite films on pure titanium. Ceram. Int..

[47] Krishnamurithy G., Yahya N.A., Mehrali M., Mehrali M., Mohan S., Murali M.R., Raghavendran H.R.B., Kamarul T. (2016). Effects of carbon doping on the microstructural, micro/nano-mechanical, and mesenchymal stromal cells biocompatibility and osteogenic differentiation properties of alumina. Ceram. Int..

[48] Koo K.N., Ismail A.F., Othman M.H.D., Bidin N., Rahman M.A. (2019). Preparation and characterization of superparamagnetic magnetite (Fe_3_O_4_) nanoparticles: A short review. Malays. J. Fundam. Appl. Sci..

[49] Yew Y.P., Shameli K., Miyake M., Khairudin N.B.B.A., Mohamad S.E.B., Naiki T., Lee K.X. (2020). Green biosynthesis of superparamagnetic magnetite Fe_3_O_4_ nanoparticles and biomedical applications in targeted anticancer drug delivery system: A review. Arab. J. Chem..

[50] Anghelache M., Turtoi M., Petrovici A.R., Fifere A., Pinteala M., Calin M. (2021). Development of dextran-coated magnetic nanoparticles loaded with protocatechuic acid for vascular inflammation therapy. Pharmaceutics.

[51] Cole A.J., David A.E., Wang J., Galbán C.J., Hill H.L., Yang V.C. (2011). Polyethylene glycol modified, cross-linked starch-coated iron oxide nanoparticles for enhanced magnetic tumor targeting. Biomaterials.

[52] Mannu R., Karthikeyan V., Velu N., Arumugam C., Roy V.A., Gopalan A.-I., Saianand G., Sonar P., Lee K.-P., Kim W.-J. (2021). Polyethylene glycol coated magnetic nanoparticles: Hybrid nanofluid formulation, properties and drug delivery prospects. Nanomaterials.

[53] Kim S.Y., Ramaraj B., Yoon K. (2012). Preparation and characterization of polyvinyl alcohol-grafted Fe_3_O_4_ magnetic nanoparticles through glutaraldehyde. Surf. Interface Anal..

[54] Bilal M., Asgher M. (2015). Sandal reactive dyes decolorization and cytotoxicity reduction using manganese peroxidase immobilized onto polyvinyl alcohol-alginate beads. Chem. Cent. J..

[55] Nafchi R.F., Ahmadi R., Heydari M., Rahimipour M.R., Molaei M.J., Unsworth L. (2022). In Vitro Study: Synthesis and Evaluation of Fe_3_O_4_/CQD Magnetic/Fluorescent Nanocomposites for Targeted Drug Delivery, MRI, and Cancer Cell Labeling Applications. Langmuir.

[56] Chen D.-Z., Tang Q., Li X., Zhou X., Zang J., Xiang J.-Y., Guo C.-Q., Xue W.-Q. (2012). Biocompatibility of magnetic Fe_3_O_4_ nanoparticles and their cytotoxic effect on MCF-7 cells. Int. J. Nanomed..

[57] Ankamwar B., Lai T., Huang J., Liu R., Hsiao M., Chen C., Hwu Y.K. (2010). Biocompatibility of Fe_3_O_4_ nanoparticles evaluated by in vitro cytotoxicity assays using normal, glia and breast cancer cells. Nanotechnology.

[58] Li Y., Liu J., Zhong Y., Zhang J., Wang Z., Wang L., An Y.-L., Lin M., Gao Z., Zhang J. (2011). Biocompatibility of Fe_3_O_4_@ Au composite magnetic nanoparticles in vitro and in vivo. Int. J. Nanomed..

[59] Stamopoulos D., Manios E., Gogola V., Niarchos D., Pissas M. (2010). On the biocompatibility of Fe_3_O_4_ ferromagnetic nanoparticles with human blood cells. J. Nanosci. Nanotechnol..

[60] Wang X.-L., Yuan X.-Z., Huang H.-J., Leng L.-J., Li H., Peng X., Wang H., Liu Y., Zeng G.-M. (2014). Study on the solubilization capacity of bio-oil in diesel by microemulsion technology with Span80 as surfactant. Fuel Process. Technol..

[61] Aydın R.S.T., Pulat M. (2012). 5-Fluorouracil encapsulated chitosan nanoparticles for pH-stimulated drug delivery: Evaluation of controlled release kinetics. J. Nanomater..

[62] Li P., Wang Y., Peng Z., She F., Kong L. (2011). Development of chitosan nanoparticles as drug delivery systems for 5-fluorouracil and leucovorin blends. Carbohydr. Polym..

[63] de Oliveira B.E., Amorim O.H.J., Lima L.L., Rezende R.A., Mestnik N.C., Bagatin E., Leonardi G.R. (2021). 5-Fluorouracil, innovative drug delivery systems to enhance bioavailability for topical use. J. Drug Deliv. Sci. Technol..

[64] Vilaça N., Amorim R., Machado A.F., Parpot P., Pereira M.F., Sardo M., Rocha J., Fonseca A.M., Neves I.C., Baltazar F. (2013). Potentiation of 5-fluorouracil encapsulated in zeolites as drug delivery systems for in vitro models of colorectal carcinoma. Colloids Surf. B Biointerfaces.

[65] Pal P., Pandey J.P., Sen G. (2018). Sesbania gum based hydrogel as platform for sustained drug delivery: An ‘in vitro’study of 5-Fu release. Int. J. Biol. Macromol..

[66] Ghiringhelli F., Apetoh L. (2015). Enhancing the anticancer effects of 5-fluorouracil: Current challenges and future perspectives. Biomed. J..

[67] Sethy C., Kundu C.N. (2021). 5-Fluorouracil (5-FU) resistance and the new strategy to enhance the sensitivity against cancer: Implication of DNA repair inhibition. Biomed. Pharmacother..

[68] Nematollahi E., Pourmadadi M., Yazdian F., Fatoorehchi H., Rashedi H., Nigjeh M.N. (2021). Synthesis and characterization of chitosan/polyvinylpyrrolidone coated nanoporous γ-Alumina as a pH-sensitive carrier for controlled release of quercetin. Int. J. Biol. Macromol..

[69] Hu Q., Luo Y. (2016). Polyphenol-chitosan conjugates: Synthesis, characterization, and applications. Carbohydr. Polym..

[70] Martínez-Mera I., Espinosa-Pesqueira M., Pérez-Hernández R., Arenas-Alatorre J. (2007). Synthesis of magnetite (Fe_3_O_4_) nanoparticles without surfactants at room temperature. Mater. Lett..

[71] Sonmez M., Georgescu M., Alexandrescu L., Gurau D., Ficai A., Ficai D., Andronescu E. (2015). Synthesis and applications of Fe_3_O_4_/SiO_2_ core-shell materials. Curr. Pharm. Des..

[72] Zhi J., Wang Y., Lu Y., Ma J., Luo G. (2006). In situ preparation of magnetic chitosan/Fe_3_O_4_ composite nanoparticles in tiny pools of water-in-oil microemulsion. React. Funct. Polym..

[73] Zhang C., Dai Y., Wu Y., Lu G., Cao Z., Cheng J., Wang K., Yang H., Xia Y., Wen X. (2020). Facile preparation of polyacrylamide/chitosan/Fe_3_O_4_ composite hydrogels for effective removal of methylene blue from aqueous solution. Carbohydr. Polym..

[74] Wulandari I.O., Mardila V.T., Santjojo D.D.H., Sabarudin A. (2018). Preparation and characterization of chitosan-coated Fe_3_O_4_ nanoparticles using ex-situ co-precipitation method and tripolyphosphate/sulphate as dual crosslinkers. IOP Conference Series: Materials Science and Engineering.

[75] Naderi Z., Azizian J. (2018). Synthesis and characterization of carboxymethyl chitosan/Fe_3_O_4_ and MnFe_2_O_4_ nanocomposites hydrogels for loading and release of curcumin. J. Photochem. Photobiol. B Biol..

[76] Ahmadi M., Pourmadadi M., Ghorbanian S.A., Yazdian F., Rashedi H. (2021). Ultra pH-sensitive nanocarrier based on Fe_2_O_3_/chitosan/montmorillonite for quercetin delivery. Int. J. Biol. Macromol..

[77] Jahanizadeh S., Yazdian F., Marjani A., Omidi M., Rashedi H. (2017). Curcumin-loaded chitosan/carboxymethyl starch/montmorillonite bio-nanocomposite for reduction of dental bacterial biofilm formation. Int. J. Biol. Macromol..

[78] Arıca B., Çalış S., Kaş H., Sargon M., Hıncal A. (2002). 5-Fluorouracil encapsulated alginate beads for the treatment of breast cancer. Int. J. Pharm..

[79] Honary S., Ebrahimi P., Hadianamrei R. (2013). Optimization of size and encapsulation efficiency of 5-FU loaded chitosan nanoparticles by response surface methodology. Curr. Drug Deliv..

[80] Rață D.M., Cadinoiu A.N., Atanase L.I., Bacaita S.E., Mihalache C., Daraba O.-M., Gherghel D., Popa M. (2019). “In vitro” behaviour of aptamer-functionalized polymeric nanocapsules loaded with 5-fluorouracil for targeted therapy. Mater. Sci. Eng. C.

[81] Jafari S., Soleimani M., Badinezhad M. (2022). Application of different mathematical models for further investigation of in vitro drug release mechanisms based on magnetic nano-composite. Polym. Bull..

[82] Hervault A., Dunn A.E., Lim M., Boyer C., Mott D., Maenosono S., Thanh N.T.K. (2016). Doxorubicin loaded dual pH-and thermo-responsive magnetic nanocarrier for combined magnetic hyperthermia and targeted controlled drug delivery applications. Nanoscale.

[83] Jarosz M., Pawlik A., Szuwarzyński M., Jaskuła M., Sulka G.D. (2016). Nanoporous anodic titanium dioxide layers as potential drug delivery systems: Drug release kinetics and mechanism. Colloids Surf. B Biointerfaces.

[84] Higuchi T. (1961). Rate of release of medicaments from ointment bases containing drugs in suspension. J. Pharm. Sci..

[85] Bruschi M.L. (2015). Strategies to Modify the Drug Release from Pharmaceutical Systems.

[86] Kawada K., Yonei T., Ueoka H., Kiura K., Tabata M., Takigawa N., Harada M., Tanimoto M. (2002). Comparison of chemosensitivity tests: Clonogenic assay versus MTT assay. Acta Med. Okayama.

[87] Nikzad S., Baradaran-Ghahfarokhi M., Nasri P. (2014). Dose-response modeling using MTT assay: A short review. Life Sci. J..

[88] Sharma N., Sharma V., Jain Y., Kumari M., Gupta R., Sharma S., Sachdev K. (2017). Synthesis and characterization of graphene oxide (GO) and reduced graphene oxide (rGO) for gas sensing application. Macromolecular Symposia.

[89] Queiroz M.F., Teodosio Melo K.R., Sabry D.A., Sassaki G.L., Rocha H.A.O. (2014). Does the use of chitosan contribute to oxalate kidney stone formation?. Mar. Drugs.

[90] Varma R., Vasudevan S. (2020). Extraction, characterization, and antimicrobial activity of chitosan from horse mussel modiolus modiolus. ACS Omega.

[91] Atangana E., Chiweshe T.T., Roberts H. (2019). Modification of novel chitosan-starch cross-linked derivatives polymers: Synthesis and characterization. J. Polym. Environ..

[92] Yasmeen S., Kabiraz M.K., Saha B., Qadir M., Gafur M., Masum S. (2016). Chromium (VI) ions removal from tannery effluent using chitosan-microcrystalline cellulose composite as adsorbent. Int. Res. J. Pure Appl. Chem..

[93] Abdel-Naby A.S., Nabil S., Aldulaijan S., Ababutain I.M., Alghamdi A.I., Almubayedh S., Khalil K.D. (2021). Synthesis, Characterization of Chitosan-Aluminum Oxide Nanocomposite for Green Synthesis of Annulated Imidazopyrazol Thione Derivatives. Polymers.

[94] Gerami S.E., Pourmadadi M., Fatoorehchi H., Yazdian F., Rashedi H., Nigjeh M.N. (2021). Preparation of pH-sensitive chitosan/polyvinylpyrrolidone/α-Fe_2_O_3_ nanocomposite for drug delivery application: Emphasis on ameliorating restrictions. Int. J. Biol. Macromol..

[95] Melo-Silveira R.F., Fidelis G.P., Costa M.S.S.P., Telles C.B.S., Dantas-Santos N., de Oliveira Elias S., Ribeiro V.B., Barth A.L., Macedo A.J., Leite E.L. (2011). In vitro antioxidant, anticoagulant and antimicrobial activity and in inhibition of cancer cell proliferation by xylan extracted from corn cobs. Int. J. Mol. Sci..

[96] Mahdaviani P., Bahadorikhalili S., Navaei-Nigjeh M., Vafaei S.Y., Esfandyari-Manesh M., Abdolghaffari A.H., Daman Z., Atyabi F., Ghahremani M.H., Amini M. (2017). Peptide functionalized poly ethylene glycol-poly caprolactone nanomicelles for specific cabazitaxel delivery to metastatic breast cancer cells. Mater. Sci. Eng. C.

[97] Sun X., Shen J., Yu D., Ouyang X.-K. (2019). Preparation of pH-sensitive Fe_3_O_4_@ C/carboxymethyl cellulose/chitosan composite beads for diclofenac sodium delivery. Int. J. Biol. Macromol..

[98] Tarlani A., Isari M., Khazraei A., Moghadam M.E. (2017). New sol-gel derived aluminum oxide-ibuprofen nanocomposite as a controlled releasing medication. Nanomed. Res. J..

[99] Liu C., Shih K., Gao Y., Li F., Wei L. (2012). Dechlorinating transformation of propachlor through nucleophilic substitution by dithionite on the surface of alumina. J. Soils Sediments.

[100] Atrak K., Ramazani A., Fardood S.T. (2018). Green synthesis of amorphous and gamma aluminum oxide nanoparticles by tragacanth gel and comparison of their photocatalytic activity for the degradation of organic dyes. J. Mater. Sci. Mater. Electron..

[101] Busca G., Lorenzelli V., Ramis G., Willey R.J. (1993). Surface sites on spinel-type and corundum-type metal oxide powders. Langmuir.

[102] Borbane S., Pande V., Vibhute S., Kendre P., Dange V. (2015). Design and fabrication of ordered mesoporous alumina scaffold for drug delivery of poorly water soluble drug. Austin Ther..

[103] Naayi S.A., Hassan A.I., Salim E.T. (2018). FTIR and X-ray diffraction analysis of Al_2_O_3_ nanostructured thin film prepared at low temperature using spray pyrolysis method. Int. J. Nanoelectron. Mater..

[104] Dabbagh H.A., Zamani M. (2011). Catalytic conversion of alcohols over alumina–zirconia mixed oxides: Reactivity and selectivity. Appl. Catal. A Gen..

[105] Abbasi Z., Haghighi M., Fatehifar E., Saedy S. (2011). Synthesis and physicochemical characterizations of nanostructured Pt/Al_2_O_3_–CeO_2_ catalysts for total oxidation of VOCs. J. Hazard. Mater..

[106] Zavareh S., Avanes A., Beiramyan P. (2017). Effective and selective removal of aromatic amines from water by Cu^2+^-treated chitosan/alumina nanocomposite. Adsorpt. Sci. Technol..

[107] Prakash B., Jothirajan M., Umapathy S., Amala V. (2013). Synthesis and characterization of biodegradable ultrasonicated films made from chitosan/Al_2_O_3_ polymer nanocomposites. Phys. Procedia.

[108] Bozorgpour F., Ramandi H.F., Jafari P., Samadi S., Yazd S.S., Aliabadi M. (2016). Removal of nitrate and phosphate using chitosan/Al_2_O_3_/Fe_3_O_4_ composite nanofibrous adsorbent: Comparison with chitosan/Al_2_O_3_/Fe_3_O_4_ beads. Int. J. Biol. Macromol..

[109] Li S., Zhang T., Tang R., Qiu H., Wang C., Zhou Z. (2015). Solvothermal synthesis and characterization of monodisperse superparamagnetic iron oxide nanoparticles. J. Magn. Magn. Mater..

[110] Zhang S., Zhang Y., Liu J., Xu Q., Xiao H., Wang X., Xu H., Zhou J. (2013). Thiol modified Fe_3_O_4_@ SiO_2_ as a robust, high effective, and recycling magnetic sorbent for mercury removal. Chem. Eng. J..

[111] Pham X.N., Nguyen T.P., Pham T.N., Tran T.T.N., Tran T.V.T. (2016). Synthesis and characterization of chitosan-coated magnetite nanoparticles and their application in curcumin drug delivery. Adv. Nat. Sci. Nanosci. Nanotechnol..

[112] Arévalo-Cid P., Isasi J., Caballero A.C., Martín-Hernández F., González-Rubio R. (2021). Effects of shell-thickness on the powder morphology, magnetic behavior and stability of the chitosan-coated Fe_3_O_4_ nanoparticles. Boletín Soc. Española Cerámica Vidr..

[113] Nivethaa E., Dhanavel S., Narayanan V., Vasu C.A., Stephen A. (2015). An in vitro cytotoxicity study of 5-fluorouracil encapsulated chitosan/gold nanocomposites towards MCF-7 cells. RSC Adv..

[114] Akmaz S., Adıgüzel E.D., Yasar M., Erguven O. (2013). The effect of Ag content of the chitosan-silver nanoparticle composite material on the structure and antibacterial activity. Adv. Mater. Sci. Eng..

[115] Omidi S., Kakanejadifard A. (2018). Eco-friendly synthesis of graphene–chitosan composite hydrogel as efficient adsorbent for Congo red. RSC Adv..

[116] Loh K.-S., Lee Y.H., Musa A., Salmah A.A., Zamri I. (2008). Use of Fe_3_O_4_ nanoparticles for enhancement of biosensor response to the herbicide 2, 4-dichlorophenoxyacetic acid. Sensors.

[117] Dai L., Jin Z., Liu X., Feng L., Ma J., Ling Z. (2021). Green Synthesis of Carbon-Encapsulated Magnetic Fe_3_O_4_ Nanoparticles Using Hydrothermal Carbonization from Rattan Holocelluloses. Coatings.

[118] Bakr E.A., El-Nahass M.N., Hamada W.M., Fayed T.A. (2021). Facile synthesis of superparamagnetic Fe_3_O_4_@ noble metal core–shell nanoparticles by thermal decomposition and hydrothermal methods: Comparative study and catalytic applications. RSC Adv..

[119] Tummala S., Kumar M.S., Prakash A. (2015). Formulation and characterization of 5-Fluorouracil enteric coated nanoparticles for sustained and localized release in treating colorectal cancer. Saudi Pharm. J..

[120] Moisescu-goia C., Muresan-Pop M., Simon V. (2017). XRD checking of crystalline forms resulted by slow evaporation of 5-fluorouracil solutions obtained with different solvents. Studia Univ. Babes Bolyai Phys..

[121] Mohammad O., Faisal S.M., Ahmad N., Rauf M., Umar M.S., Mujeeb A.A., Pachauri P., Ahmed A., Kashif M., Ajmal M. (2019). Bio-mediated synthesis of 5-FU based nanoparticles employing orange fruit juice: A novel drug delivery system to treat skin fibrosarcoma in model animals. Sci. Rep..

[122] Samadi A., Pourmadadi M., Yazdian F., Rashedi H., Navaei-Nigjeh M. (2021). Ameliorating quercetin constraints in cancer therapy with pH-responsive agarose-polyvinylpyrrolidone-hydroxyapatite nanocomposite encapsulated in double nanoemulsion. Int. J. Biol. Macromol..

[123] Rahimi M., Safa K.D., Alizadeh E., Salehi R. (2017). Dendritic chitosan as a magnetic and biocompatible nanocarrier for the simultaneous delivery of doxorubicin and methotrexate to MCF-7 cell line. New J. Chem..

[124] Caputo F., Clogston J., Calzolai L., Rösslein M., Prina-Mello A. (2019). Measuring particle size distribution of nanoparticle enabled medicinal products, the joint view of EUNCL and NCI-NCL. A step by step approach combining orthogonal measurements with increasing complexity. J. Control. Release.

[125] Honary S., Zahir F. (2013). Effect of zeta potential on the properties of nano-drug delivery systems-a review (Part 2). Trop. J. Pharm. Res..

[126] Zavareh H.S., Pourmadadi M., Moradi A., Yazdian F., Omidi M. (2020). Chitosan/carbon quantum dot/aptamer complex as a potential anticancer drug delivery system towards the release of 5-fluorouracil. Int. J. Biol. Macromol..

[127] Wang R., Song B., Wu J., Zhang Y., Chen A., Shao L. (2018). Potential adverse effects of nanoparticles on the reproductive system. Int. J. Nanomed..

[128] Kazemi S., Pourmadadi M., Yazdian F., Ghadami A. (2021). The synthesis and characterization of targeted delivery curcumin using chitosan-magnetite-reduced graphene oxide as nano-carrier. Int. J. Biol. Macromol..

